# Strengthening and Embrittling Mechanism of Super 304H Steel during Long-Term Aging at 650 °C

**DOI:** 10.3390/ma17030740

**Published:** 2024-02-03

**Authors:** Yue Wu, Fufangzhuo Chai, Junjian Liu, Jiaqing Wang, Yong Li, Chengchao Du

**Affiliations:** 1 Datang Boiler Pressure Vessel Inspection Center Co., Ltd., Hefei 230088, China; 2 East China Electric Power Test and Research Institute, China Datang Corporation Science and Technology Research Institute Co., Ltd., Hefei 230088, China; 3 School of Materials Science and Engineering, Jiangsu University, Zhenjiang 212013, China

**Keywords:** super 304H, aging treatment, precipitate, hardness, impact toughness

## Abstract

Super 304H has been a crucial material for ultra-supercritical boilers. However, the relationship between microstructure evolution, strengthening mechanism, and embrittling behavior during long-term aging was lacking investigation. This investigation aimed to reveal the strengthening and embrittling mechanism from precipitates in Super 304H. The results showed that the hardness increment came from the grain boundary’s M_23_C_6_ (GB’s M_23_C_6_) and intragranular nano Cu-rich particles. After being aged for 5000 h, the GB’s M_23_C_6_ and nano Cu-rich particles provided a hardness increment of approximately 10 HV and 30 HV, respectively. The impact toughness gradually decreased from 213 J/cm^2^ to 161 J/cm^2^ with the extending aging time. For the aged Super 304H, the GB’s M_23_C_6_ provided a higher cracking source. In addition, the nano Cu-rich particle restricted the twin-induced plastic deformation of austenitic grain and depressed the absorbed energy from austenitic grain deformation.

## 1. Introduction

Ultra-supercritical boilers (USCBs) have been widely employed to improve the thermal efficiency of power plants and reduce CO_2_ emissions [1,2,3]. With the increase in temperature and pressure of USCBs, the mechanical properties of heat-resistant steels have been greatly improved. Among them, the development of Super 304H is a typical example. The Super 304H steel was developed from Type 304H steel by adding 3% Cu, 0.3% Nb, and 0.1% N. Generally, the Nb combining with C and N generates the Nb(C,N) (MX) phase in FCC-typed steel [4,5,6]. Part of MX phase are nanoscale particles after solid solution treatment which contributes to the fine grain of Super 304H. The Cu is solid solution atom in the Super 304H after solid solution treatment. During the long-term service, Cu precipitates and forms a nanoscale Cu-rich particle [7,8,9,10]. The nanoscale MX particle and Cu-rich particle greatly strengthen the creep performance of Super 304H [11,12,13].

Impact toughness is a key factor for austenitic heat-resistant steel, especially in high-temperature environments such as supercritical boilers and petrochemical equipment. Under these conditions, excellent impact toughness is essential to ensure the material’s resistance to crack propagation and brittle fracture. However, the austenitic heat-resistant steel shows a lower impact toughness after aging treatment. Wang et al. [14] indicated that the impact toughness of 25Cr–20Ni–Nb–N (HR3C) decreased to approximately 40 J/cm^2^ with an aging time longer than 1000 h at a temperature between 650 °C and 750 °C. Bai et al. [15] revealed that the impact toughness of HR3C was below 50 J/cm^2^ after being aged for 3000 h at 700 °C. Zielinski et al. [16] also observed the poor impact toughness below 50 J/cm^2^ after aging for 1000 h. The fractured surface of the aged HR3C showed a character of intergranular fractures [17,18]. The embrittlement mechanism of the HR3C was the brittle carbides on the grain boundary which reduced the strength of the grain boundary. In addition, the impact toughness of the austenitic heat-resistant steel of 22Cr25Ni3W3CuCoNb (Sanicro25) was only approximately 30 J/cm^2^ after being aged for 200 h at 700 °C [19]. It also showed an intergranular fracture surface after an aging time longer than 200 h. The embrittlement of aged Sanicro25 was mainly attributed to the brittle precipitates on the grain boundary.

For Super 304H, the impact toughness was greatly improved compared with that of HR3C and Sanicro25. The impact toughness of Super 304H after solid solution treatment was approximately 210 J/cm^2^. After heat treatment for 5000 h at 650 °C, the impact toughness only declined to approximately 160 J/cm^2^ [20]. According to the dimples on the fractured surfaces, the Super 304H also showed a ductile fracture character after aging treatment. Until now, the embrittlement mechanism was still unclear. The effect of microstructure evolution on the strength and impact toughness of the Super 304H steel during aging treatment needs to be systematically investigated.

## 2. Materials and Experiments

The composition of the examined Super 304H steel was as follows: 0.11 C, 0.21 Si, 0.35 Mn, 18.2 Cr, 9.1 Ni, 2.9 Cu, 0.51 Nb, 0.08 N, and 0.03 P (wt%, bal. Fe). The Super 304H was annealed at 1150 °C for 1 h after rolling. A muffle furnace was used to heat-treat the Super 304H specimen in ambient air. The Super 304H steel was aged for 1000 h, 2000 h, and 5000 h at 650 °C. After aging treatment, the samples with a dimension of 10 mm × 10 mm × 55 mm were machined for Charpy V-notch impact testing at room temperature. The maximum impacting speed was approximately 5.4 m/s. After impacting test, the fractured surfaces were observed using a scanning electron microscope (SEM, FEI Nova 450) under second electron imaging (SEI) mode. The microstructure of the original and aged Super 304H was observed using SEM (FEI Nova 450 under SEI mode with acceleration voltage of 10 kV and work distance of 12 mm, JSM 7900F under backscattered electron imaging (BEI) mode with acceleration voltage of 3 kV and work distance of 8 mm) after a final polish with nano SiO_2_ suspension and etching using FeCl_3_+HCl+H_2_O solution. The Vickers hardness test was conducted using the FM-ARS900 hardness tester, employing a 1.96 kgf (19.208 N) indenter. Five measurements were taken, and the results were averaged for accuracy. The thin foil of the Super 304H aged for 5000 h and the thin foil near the fractured surface of original Super 304H were prepared using ion thinning (Gatan PIPS II) and observed using a transmission electron microscope (TEM, JEOL JEM-2100 PLUS). The phase diagram of Super 304H was calculated using JMatPro 6.0.

## 3. Results and Discussion

### 3.1. Microstructure Evolution during Long-Term Aging

**Figure 1:**
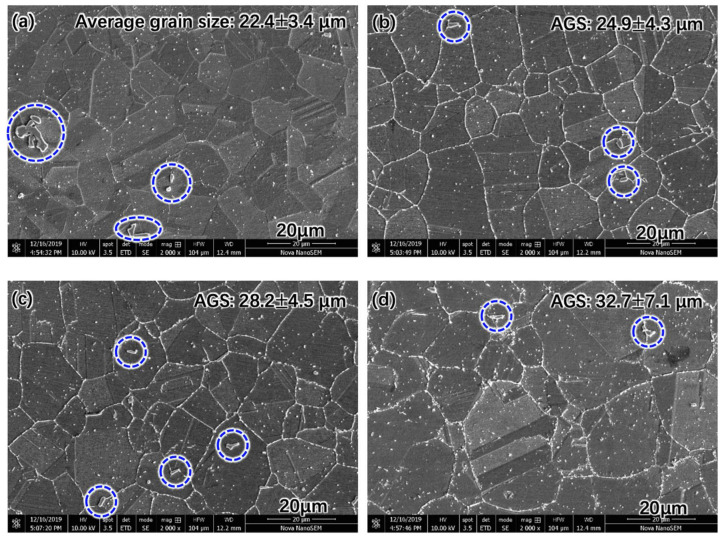
Grain evolution during aging treatment at 650 °C: (**a**) original, (**b**) 1000 h, (**c**) 2000 h, (**d**) 5000 h. (The coarse precipitates indicated by the blue circles in Figure 1 were primary MX phase).

The microstructure of the Super 304H with different aging times is shown in Figure 1. It was observed that the grain size of the original Super 304H was approximately 22.4 μm. After being aged for 5000 h at 650 °C, the grain size gradually increased to approximately 32.7 μm as shown in Figure 1d. In general, the grain growth during aging treatment was insignificant. The precipitates were also distinguished in Figure 1. The coarse precipitates indicated by the blue circles in Figure 1 were primary MX phase. The coarser MX phase was generated during the high temperature softening process at 1270 °C during the heat treatment. The fine precipitates (light particles) inside the grain were also MX phase. The fine precipitate generated during the solid solution treatment at 1100 °C during the heat treatment. During the aging process, the precipitates mainly generated on the grain boundary, shown in Figure 1b–d.

**Figure 2:**
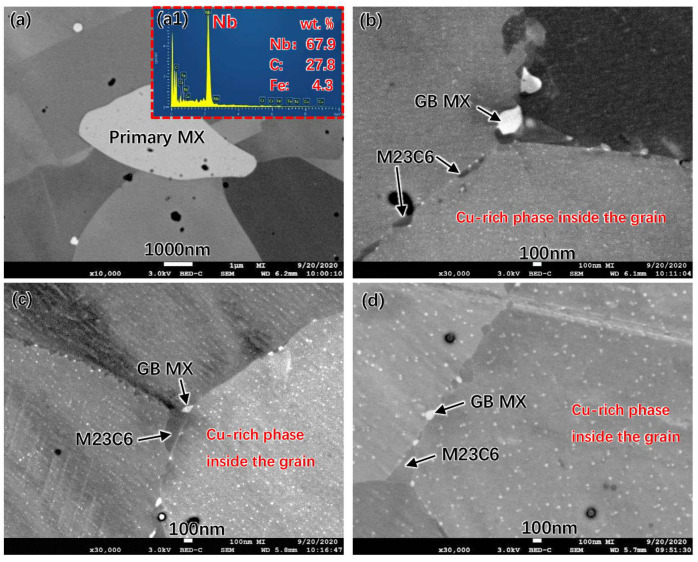
Precipitates evolution during aging treatment at 650 °C: (**a**) Original, (a1) EDS pattern of MX phase, (**b**) 1000 h, (**c**) 2000 h, (**d**) 5000 h.

In order to investigate the precipitates near the grain boundary, the magnified SEM images were captured as shown in Figure 2. From Figure 2a, only coarser primary MX phase the fine intragranular MX particle was observed. After aging treatment, the M_23_C_6_ particle and grain boundary’s MX phase was observed as shown in Figure 2b–d. In addition, the fine Cu-rich phase gradually generated inside the grain.

**Figure 3:**
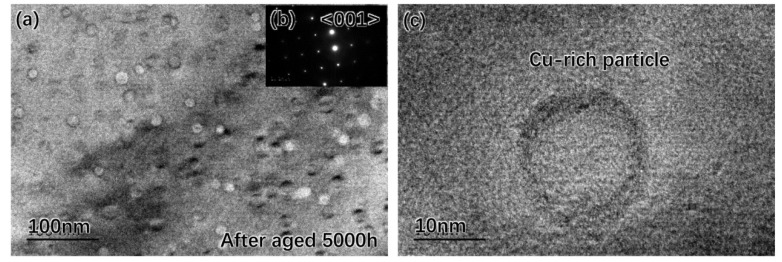
Cu-rich particle after aging for 5000 h: (**a**) Cu-rich particles inside the grain, (**b**) SAED pattern, (**c**) morphology of a single Cu-rich particle.

The nano Cu-rich phase in the Super 304H after being aged for 5000 h is shown in Figure 3. The Cu-rich phase was observed under the crystal axis of <001> as shown in Figure 3b. The magnified Cu-rich phase is shown in Figure 3c. The diameter of the nano Cu-rich phase was approximately 15 nm. It was concluded that the Cu-rich particle had a coherent relationship with the FCC matrix according to the single set of SAED spots in Figure 3b.

**Figure 4:**
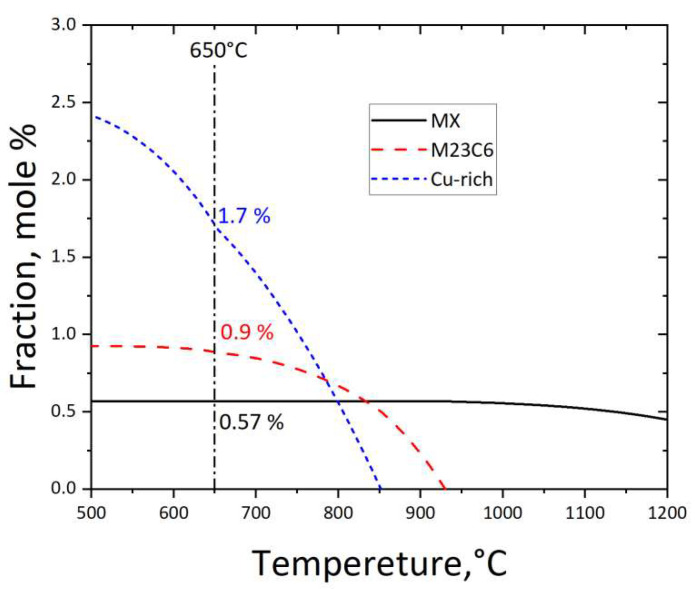
Phase diagram of Super 304H between 500 h and 1200 h.

The phase fraction of the Super 304H as a function of temperature is shown in Figure 4. It was observed that the fraction of MX at the temperature of 1100 °C and 650 °C was 0.5% and 0.57%, respectively. This meant that the MX phase would gradually increase with a volume fraction of 0.07% during aging treatment. In this investigation, the MX phase gradually generated on the grain boundary. The volume fraction of M_23_C_6_ and the Cu-rich phase at the temperature of 650 °C was 0.9% and 1.7%, respectively. The M_23_C_6_ gradually generated on the grain boundary during aging treatment.

### 3.2. Hardness and Impact Toughness Evolution during Long-Term Aging

**Figure 5:**
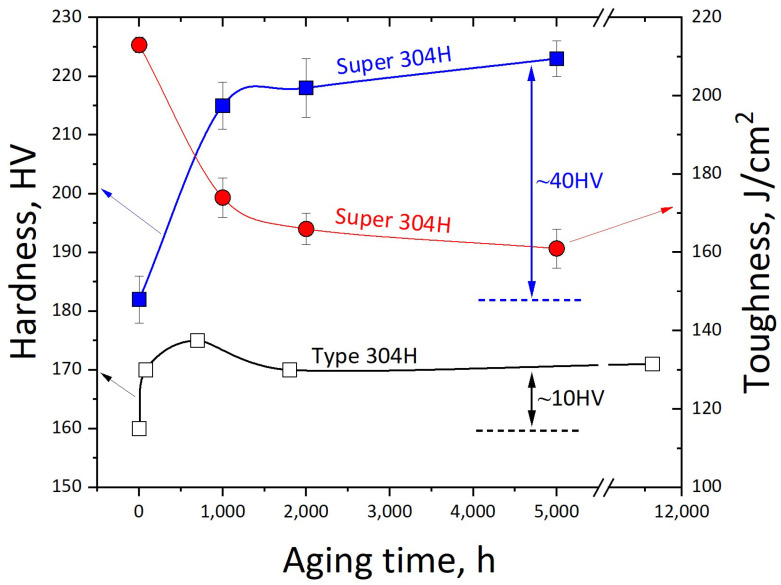
Hardness and impact toughness of Super 304H as a function of aging time.

The hardness and impact toughness of the Super 304H are shown in Figure 5. Regarding the hardness, the original Super 304H was approximately 182 HV. After being aged for 1000 h, the hardness increased to approximately 215 HV. When the aging time increased to 5000 h, the hardness increased to 223 HV. The hardness increment of the type 304H is also plotted in Figure 5. The hardness of the original type 304H was 160 HV. After aged for 10,000 h, the hardness increased to 171 HV [21]. The hardness variation of the Super 304H and type 304H came from the different strengthening mechanisms. This will be discussed in Section 3.3.

**Figure 6:**
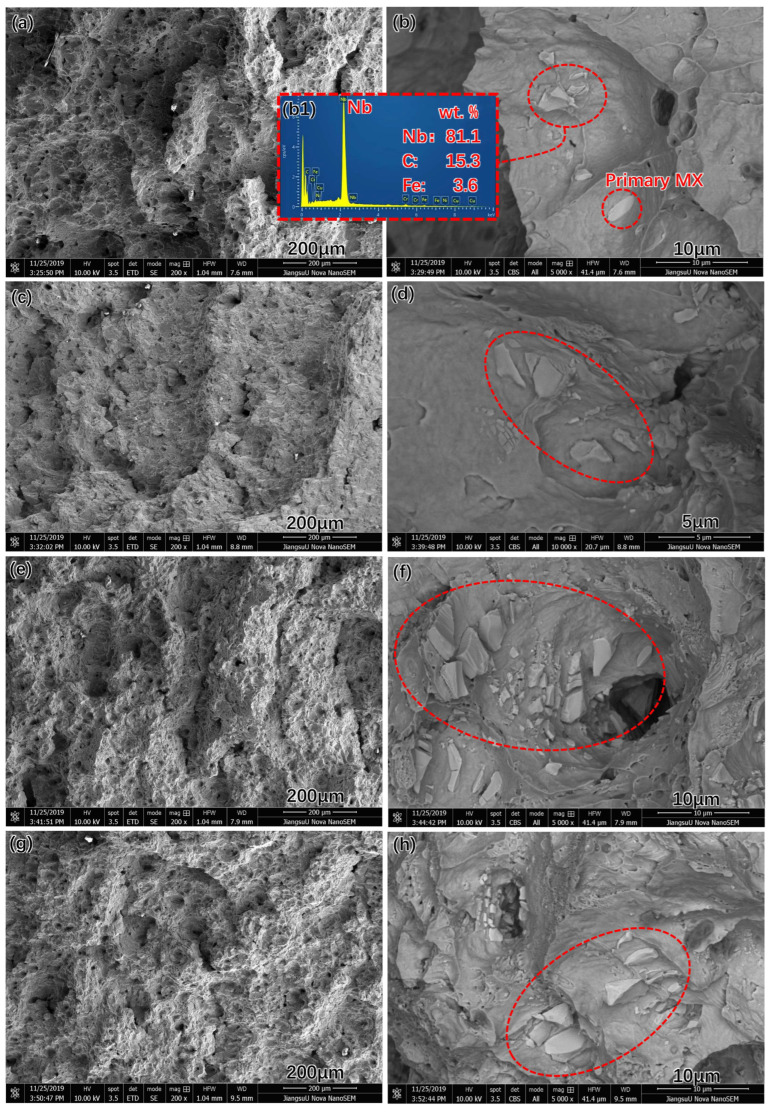
Fractured surfaces of Super 304H: (**a**,**b**) original, (b1) EDS pattern of MX phase, (**c**,**d**) 1000 h, (**e**,**f**) 2000 h, (**g**,**h**) 5000 h. (the brittle fractured primary MX phase was observed on the fractured surfaces as indicated in red circles as shown in Figure 6b,d,f,h).

The impact toughness of the Super 304H gradually declined as a function of aging time. The original impact toughness of the Super 304H was approximately 213 J/cm^2^. After being aged for 1000 h, the impact toughness declined to approximately 173 J/cm^2^. When the aging time extended to 5000 h, the impact toughness declined to approximately 161 J/cm^2^. The fractured surface of the Super 304H with different aging times is shown in Figure 6. According to the Figure 6a,c,e,g, the character of ductile fracture was observed. After magnification, the brittle fractured primary MX phase was observed on the fractured surfaces as indicated in red circles as shown in Figure 6b,d,f,h. The fractured MX phase was common on the fractured surface of Super 304H steel [22]. The fraction and size of the primary MX phase was stable during aging treatment. Therefore, it was not the influence factor of impact toughness decline during aging treatment. The embrittlement of the Super304H will be discussed in Section 3.4.

### 3.3. Strengthening Mechanism Evolution during Long-Term Aging

#### 3.3.1. Solid Solution

For the original Super304H, the atoms of C and Cu were dissolved in the FCC-typed matrix. The solid strengthening effect mainly came from the two elements. During long-term aging, the C precipitated in M_23_C_6_. As the Cu precipitated in the Cu-rich phase, the solid solution strengthening effect gradually declined with the extending of aging time.

#### 3.3.2. Precipitates

The precipitate in the original Super304H was the primary MX particle. During aging treatment, the M_23_C_6_ gradually generated on the grain boundary. According to Ref. [21], the precipitate of M_23_C_6_ strengthened the type 304H steel and only contributed to a hardness increment of 10 HV after being aged for 5000 h. The hardness increment of type 304H was a comprehensive result from solid solution strengthening effect decline and precipitating strengthening effect improvement. In this investigation, the strength from M_23_C_6_ was also evaluated as 10 HV in Super 304H after being aged for 5000 h.

Except for the 10 HV from M_23_C_6_, the additional hardness increment of 30 HV, as shown in Figure 5, could be due to the precipitating strengthening effect from Cu-rich phase [20,23]. The intragranular nano particle has a strengthening effect on the alloys [24,25,26,27]. Du et al. [28] revealed that the precipitating strengthening effect of nano Cu-rich particles depended on three parts, including order strengthening, coherency strengthening, and modulus strengthening, as shown in Equations (1)–(3):

(1) $$∆\sigma_{o}=0.81M\left(\frac{\gamma_{apb}}{2b_{M}}\right){\left(\frac{3\pi f}{8}\right)}^{0.5}$$

(2) $$∆\sigma_{c}=2.6M{\left(G_{M}\varepsilon \right)}^{1.5}{\left(\frac{rf}{0.18G_{M}b_{M}}\right)}^{0.5}$$

(3) $$∆\sigma_{m}=0.0055M{\left(∆G\right)}^{1.5}{\left(\frac{2f}{G_{M}b_{M}^{2}}\right)}^{0.5}$$

where $\gamma_{apb}$ is the anti-phase boundary energy of precipitates, $G_{M}$ is the shear modulus of the matrix, $b_{M}$ is the magnitude of the Burgers vector of the matrix, *f* is the volume fraction of precipitate, ε is the lattice–parameter mismatch, r is the mean precipitate radius, and ∆G is the shear modulus mismatch between matrix and the precipitate. The values of $∆\sigma_{c}$ and $∆\sigma_{m}$ greatly depend on the value of ε and ∆G. The values of the parameters are listed in Table 1.

**Figure 7:**
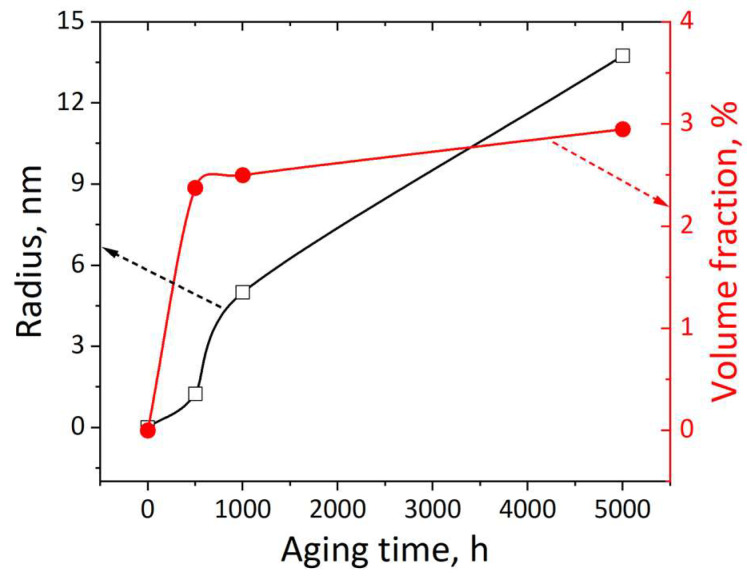
Radius and volume fraction of the Cu-rich phase as a function of aging time at 650 °C [10].

According to Ref. [10], the radius and volume fraction of the Cu-rich phase as a function of aging time is shown in Figure 7. It was observed that the radius of the Cu-rich phase gradually increased as the increase of aging time. For the volume fraction, it was rapidly increased during the short-term (500 h) aging treatment. For the aging time between 500 h and 5000 h, the volume fraction only increased 0.575 vol.%. In our work, the radius and volume fraction of the Cu-rich particles of the Super 304H aged for 5000 h were approximately 14 nm and 2.75 vol.%, respectively. The results were very close to that of Ref. [10]. In order to calculate the stress from order strengthening, coherency strengthening, and modulus strengthening, the radius and volume fraction of the Super 304H after ageing for 1000 h, 2000 h, and 5000 h are listed in Table 2. The values of $∆\sigma_{o}$, $∆\sigma_{c}$, and $∆\sigma_{m}$ are calculated and also listed in Table 2. It was observed that the stress from coherency strengthening was between 100 MPa and 181 MPa. It was the major strengthening effect among the three aspects. Therefore, the hardness increment of 30 HV was mainly produced by coherency strengthening in Super 304H.

### 3.4. Embrittling Mechanism during Long-Term Aging

**Figure 8:**
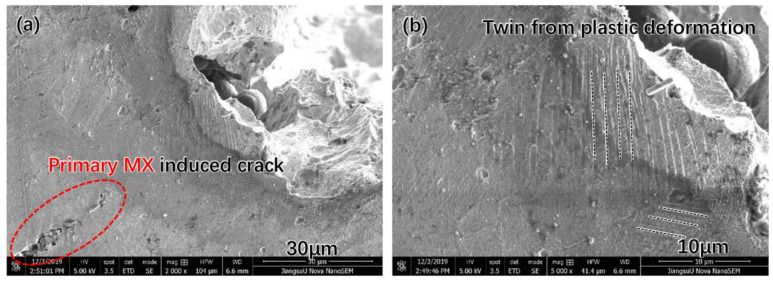
Cross-section of fractured original Super 304H: (**a**) crack near the fractured surface, (**b**) twin near the fractured surface.

**Figure 9:**
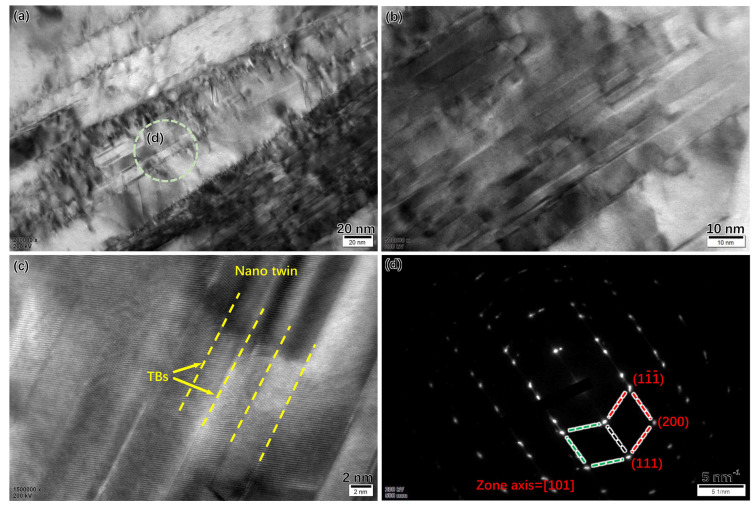
(**a**–**c**) Morphologies of nano twin near the fractured surface at different scales of original Super 304H, (**d**) SAED pattern of nano twin near the fractured surface of original Super 304H. (The circle was the region for SAED).

The microstructure of the cross-section of the original Super 304H steel near the fractured surface is shown in Figure 8. In Figure 8a, the fracture of the primary MX phase was observed. In addition, the morphology of twin deformation was observed inside the austenite grain as shown in Figure 8b. The TEM thin foil sample of near the fracture surface was carefully prepared. The microstructure of the Super 304H is shown in Figure 9. The nano twin was observed inside the grain as shown in Figure 9a, b, and c. The width of the nano twin was approximately 4 nm. The SAED image of the nano twins in the circle of Figure 9a is shown in Figure 9d.

The formation of the twin was attributed to the deformation of the grain near the fractured surface. During the impacting test, a large tensile stress was generated in the original Super 304H. The tensile stress induced the plastic deformation of the Super 304H. As the enhancing of plastic deformation, the deformation mechanism of Super 304H gradually transformed into the twinning from dislocation movement. When the stress increased to a certain extent, the fracture of the primary MX phase occurred. The fracture of the MX phase resulted in the formation of micro-cracks. Under the tensile stress in the Super 304H steel, the micro-crack propagated deeply and resulted in the fracture of the original Super 304H steel.

**Figure 10:**
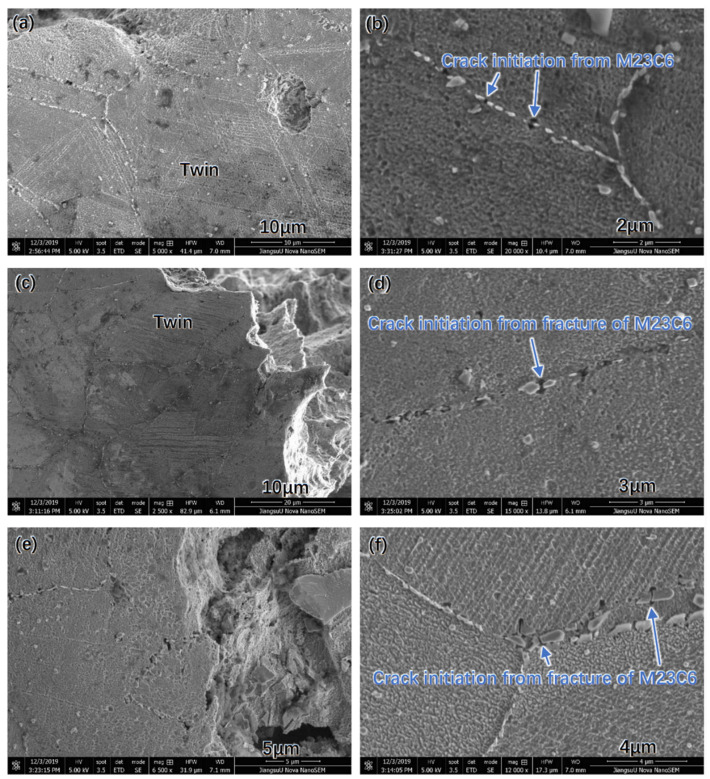
Cross-section of the fractured aged Super 304H: (**a**,**b**) 1000 h, (**c**,**d**) 2000 h, (**e**,**f**) 5000 h.

The microstructure of the cross-section of the aged Super 304H steel near the fractured surface is shown in Figure 10. It was observed that twin was formed inside the austenite grain as shown in Figure 10a,c,e. In addition, the fractured grain boundary M_23_C_6_ was observed as shown in Figure 10b,d,f. The micro-cracks were clearly observed in the M_23_C_6_. During the impacting test, the severe plastic deformation induced the twin inside the austenite grain. When the stress increased to a certain extent, the fracture of the primary MX phase and the grain boundary M_23_C_6_ occurred. The fracture of the MX phase and the grain boundary M_23_C_6_ resulted in the formation of micro-cracks. Under the tensile stress in the Super 304H steel, the micro-crack propagated deeply and resulted in the fracture of original Super 304H steel.

Previous studies had established that pre-existing deformation twins, particularly in other FCC alloy systems, can effectively enhance strength while maintaining ductility [31]. However, activating twinning proves challenging, and it typically functions as an intermediate deformation mode due to the considerably higher stress required compared to dislocation slip. Narita and Takamura highlighted the critical resolved shear stress needed for twinning [32]:

(4) $$\tau_{T}=\gamma_{SF}/(Kb_{s})$$

where $\tau_{T}$ is the critical resolved shear stress (CRSS), $\gamma_{SF}$ is the stacking fault energy, and K is a parameter which could be approximately determined to be 2. $b_{s}$ is the Burgers vector of the Shockley partial dislocation; for an FCC crystal it is a[211] = 6. For the current Super 304H, the $\gamma_{SF}$ and $b_{s}$ were established as 17 mJ/m^2^ and 0.147 nm, thus the CRSS value was estimated to be 57.8 MPa. The Cu alloy had a higher $\gamma_{SF}$ compared with that of Super 304H. The $\gamma_{SF}$ and $b_{s}$ of the nano Cu-rich particles were established as 70 mJ/m^2^ and 0.148 nm, thus the CRSS value was estimated to be 236.5 MPa. Therefore, the twinning in the Cu-rich phase was harder than that in the FCC–Fe matrix. In other words, the Cu-rich particle hindered the formation of twinning in the FCC matrix due to its higher CRSS. It could be concluded that the deformation ability of the FCC matrix with nano Cu-rich particles was depressed. The absorbed energy from plastic deformation during the impacting test was restricted. In addition, the hardened FCC matrix also helped the crack propagation during impacting test.

**Figure 11:**
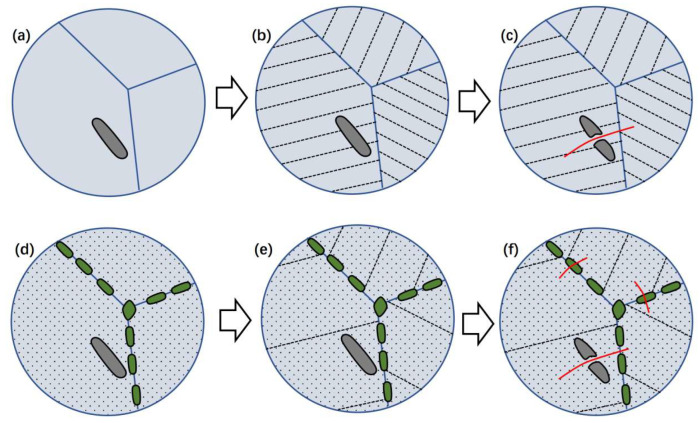
Fractured mechanism of Super 304H: (**a**–**c**) original, (**d**–**f**) aged.

Based on the above results and discussion, the embrittling of the Super304H was concluded as in Figure 11. For the Super304H after solid solution treatment, the phases were austenite and the primary MX phase. During the impacting test, the austenitic grain experienced severe plastic deformation and formed the nano twin inside the grain. When the stress increased to the cracking stress of primary MX phase, the brittle fracture occurred in the primary MX phase. The micro-crack in the primary MX phase contributed to the stress concentration in the austenite and resulted in the cracking of nearby austenite as shown in Figure 11a–c. The fracture of the solid solution treated Super304H was mainly in a ductile mode. The brittle fracture of the primary MX phase only slightly reduced the impact toughness of the Super304H as its minor volume fraction.

After aging treatment, the M_23_C_6_ and nano Cu-rich particles were generated on the grain boundary and inside the grain, respectively. The nano Cu-rich phase strengthened the austenite and declined the plastic deformation ability. During the impacting test, the plastic deformation generated in the austenitic grain first. It induced the formation of twin of austenite. With the strengthening effect of nano Cu-rich particle, the deeply twinning deformation was restricted. When the stress increased to the critical level, the fracture of the primary MX phase and grain boundary M_23_C_6_ occurred. The micro-crack in the primary MX phase and grain boundary M_23_C_6_ rapidly propagated in the hardened austenitic grain. The brittle fracture of the primary MX phase and grain boundary M_23_C_6_ provided more cracking sources for the aged Super 304H. In addition, the austenite hardened by the nano Cu-rich particles absorbed smaller energy via plastic deformation.

## 4. Conclusions

In this work, the microstructure evolution and embrittlement of Super 304H during long-term aging at 650 °C was investigated. The major conclusions were derived.

(1) The as-received Super 304H exhibited the microstructure of austenite and primary MX phase. After aging at 650 °C, the intragranular nano Cu-rich particles gradually precipitated from the austenite matrix. The particle size and volume fraction gradually increased with the extended aging time. The M_23_C_6_ precipitated on the grain boundary.
(2) The hardness gradually increased with the extending aging time. The hardness increment was approximately 40 HV after being aged for 5000 h. The hardness increase depended on the M_23_C_6_ and nano Cu-rich particles together. For the M_23_C_6_, it provided a hardness increment of approximately 10 HV. The nano Cu-rich particle provided a hardness increment of approximately 30 HV after being aged for 5000 h. The major strengthening mechanism of nano Cu-rich particle was coherency strengthening.
(3) The impact toughness gradually decreased from 213 J/cm^2^ to 161 J/cm^2^ with the extended aging time. For the aged Super 304H, the precipitated M_23_C_6_ provided a greater cracking source. In addition, the nano Cu-rich particle restricted the plastic deformation from TWIP. The absorbed energy from austenite grain deformation was also depressed. Therefore, the impact toughness gradually decreased.

## Figures and Tables

**Table 1:** Values of parameters in Equations (1)–(3) [29,30].

Parameters	Value	Parameters	Value
*M*	3.06	G_M_	73.2, GPa
*r*_apb_	0.12, J/m^2^	*ε*	0.005
*B*	0.255, nm	Δ*G*	10, GPa

**Table 2:** Stress from Equations (1)–(3) of super 304H.

Aging Time, h	*r*, nm	*f*, %	Δ*σ_o_*, MPa	Δ*σ_c_*, MPa	Δ*σ_m_*, MPa
1000	5.01	2.51	100.3	204.5	55.5
2000	7.18	2.68	103.6	253.0	57.3
5000	13.75	2.95	108.7	367.3	60.1

## Data Availability

All relevant data are within the paper.
